# Developing home cleaning intervention through community engagement to reduce infections and antimicrobial resistance in Ghanaian homes

**DOI:** 10.1038/s41598-023-37317-4

**Published:** 2023-06-28

**Authors:** Emmanuel Tsekleves, Dziedzom de Souza, Roger Pickup, Collins Ahorlu, Andy Darby

**Affiliations:** 1 ImaginationLancaster, Lancaster University, Lancaster, UK; 2 Noguchi Memorial Institute for Medical Research, University of Ghana, Accra, Ghana; 3 Biomedical and Life Sciences, Lancaster University, Lancaster, UK

**Keywords:** Antimicrobials, Bacteria, Public health, Patient education, Epidemiology

## Abstract

Globally Antimicrobial Resistance (AMR) constitutes a health crisis, particularly in developing countries, where infectious disease are commonly fatal. There is clear evidence for microbial exposure and infection transmission within the home. Personal and environmental hygiene are the best ways of reducing household infections thus decreasing the need for antibiotics and consequently diminishing AMR. Despite this being an obvious step, research efforts to understand the home environment and its impact on AMR, cleaning and possible interventions on household cleaning are limited. We combined design and microbiology methods in an innovative mixed-method approach. A traditional survey design (n = 240), a design ethnography (n = 12), a co-design workshop and a pre-intervention microbiological dust sample analysis was undertaken to provide insights for codesign workshops in which new cleaning practices might be developed to minimise any AMR bacteria present in the household environments located in the Greater Accra Region of Ghana. Microbiological analysis of household dust showed that 36.6% of bacterial isolates detected were found to carry at least one resistance to the panel of antibiotics tested. Four scenarios were generated from an economic segmentation of the survey data. 50 ethnographic insights were ‘presented’ and descriptions of 12 bacteria species that showed resistance to one or more antibiotics (representing 176 bacterial isolates that showed resistance to one or more antibiotics found in the dust samples) were presented to the participants in a codesign workshop. An intervention, a new regime of cleaning practices agreed through the co-design workshop and practiced for thirty days, was made in (n = 7) households. The high prevalence of multidrug resistance observed in this study indicate the need for antibiotics surveillance program, not only in hospital settings but also in the household environment. There is, thus, an urgent need for targeting of interventions at the household level. Activating knowledge through community engagement in the research helps in increasing public perception and breaking down the scientist-public barrier.

## Introduction

Antimicrobial resistance (AMR) is a complex global challenge driven by diverse factors cutting across disciplines. It impacts both human and animal health, as well as agriculture and the environment^1^. To combat its negative consequences new transdisciplinary approaches are needed.

Antimicrobials play a crucial role not only in maintaining human health, but also in promoting animal welfare and productivity, which ultimately contributes to global food security and safety^2^. The rising global demand for meat, poultry, and egg production resulting from population growth, however, creates an environment conducive to the spread of zoonotic diseases from animals to humans, as well as the acceleration of antimicrobial resistance^3^.

The overuse and misuse of antibiotics in animals have been identified as a significant factor in the promotion of AMR in humans. The use of antibiotics in agriculture, for instance, results in human exposure to AMR^4^ and poses serious risks to both animal and human health and welfare^5^. Upon undergoing antimicrobial treatment, humans, plants, and animals do not fully metabolize the substances, leading to their residues being excreted and entering the food chain or the environment, thereby compromising both food safety and the environment^6^. While antimicrobial residues may not be potent enough to destroy microbes, they may induce enough stress or selection to cause resistance to develop, hence exacerbating AMR. Poor hygiene, animal husbandry, water quality, and sanitation practices can also contribute to the spread of AMR^1,7^.

Bacteria found in the natural and built environment (e.g., homes, schools, hospitals, etc.) are building their resistance to drugs. They are changing to protect themselves against antibiotics they encounter. As bacteria build their resistance to antibiotics, minor cuts and infections may become life-threatening^8^. Globally AMR constitutes a health crisis, particularly in developing countries, where infectious disease commonly leads to fatalities^9^. While Ghana is committed to the global action plan to reduce AMR^10^ most attention is focussed on hospital/healthcare environments, despite evidence of microbial exposure and infection transmission in the home^11^. As a consequence, there has been little research into further understanding the domestic phenomena^12^, this is in part due to the difficulties of conducting detailed studies in household environments^13^.

Evidence collectively suggests that personal and environmental hygiene reduces the spread of infection^14^ and remains the most important cornerstone in the control of infectious disease in the home^7,15^. The environment is closely linked to the lifestyles we adopt and our cleaning practices^16^ playing a significant role in terms of microbial exposure and infection (viral/bacterial) transmission^17,18^. Although the transmission routes of dust in the home environment are well known^11^, what has not been studied is how to prevent bacterial infection in home environments and thereby reduce resistance.

The absence of a clear hygiene indicator in modern environments has caused people to be unsure about the true dangers present, leading to confusion regarding the nature of real threats such as bacterial infections, which remain invisible^19^. This has led to a more superficial approach to household cleaning, with speed and aesthetic factors more important than hygiene and disease prevention at a deeper level^20,21^.

In Ghana, hygiene guidelines are predominantly focused on hand washing^22^. However, guidance to tackle bacterial pathogens that are specific to different household environments, and which are also appropriate for people from diverse educational and cultural backgrounds, does not exist.

AMR is carried by both infectious and non-infectious bacteria with the latter carrying the potential to transfer resistance to originally AMR-sensitive infectious bacteria^23,24^. The Dust Bunny project’s specific aim was to develop an understanding of the home as a potential source of infection from AMR bacteria carried by dust by exploring hygiene practices across different household environments in Ghana. The project had the ultimate goal of reducing bacterial infection in the household environment in order to reduce AMR and to convey this message to householders. In adopting these aims, the Dust Bunny project’s aspirations were aligned to Sustainable Development Goal 3, target 3d^25^, and its desire to strengthen the capacity of a developing country in making risk reduction interventions in response to a global health issue. In this paper we present the steps to design interventions in the home through an understanding of the ‘invisible’ presence of AMR.

## Methods

To begin to address the problem a collaboration between researchers from the Lancaster University (LU), UK and the Noguchi Memorial Medical Research Institute (NMIMR), Ghana, developed the Dust Bunny project. The academic disciplines of the core research team included design (LU), microbiology (LU/NMIMR) and cultural/social epidemiology (NMIMR).

The Dust Bunny project combined design and microbiology methods in an innovative mixed-method approach. A traditional survey design, a design ethnography, a co-design workshop and a microbiological analysis provided insights for codesign workshops in which new cleaning practices were developed to minimise any AMR bacteria present in the household environments undertaken across different domestic environments (i.e., urban vs rural, private vs communal dwellings) and a range of social scales within Accra, Ghana. The procession of methods from survey instrument to design ethnography and cultural probe, made in parallel with a microbiological analysis, was designed to provide a range of insights into contemporary cleaning practices in Accra. These insights were then used by participants to codesign, in a one-day workshop, ‘new’ cleaning practices for a thirty-day intervention, which was followed by another round of microbiological analysis.

### Survey

A cross-sectional retrospective survey design was selected to provide a general description of current practices and perceptions of cleanliness and hygiene in relation to household dust and household environments. The initial questionnaire was developed at LU with input from NMIMR and, following pretesting of a paper version in a field environment and revisions, a bespoke iOS application to run the survey instrument was developed at LU and deployed online ready for use in the field. The application was developed on the iOS platform mainly due to accessibility reasons, as the NMIMR had access only to iPad devices. Thereafter, four NMIMR data collectors conducted the survey questionnaire (n = 240) in Adentan Municipal Assembly, Ga East Municipal Assembly, and La-Nkwantanang Madina Municipal Assembly. Finally, a data scientist at NMIMR provided detailed analysis of the survey results.

### Design ethnography

A design ethnography^26^ approach was developed and carried out in volunteer households (n = 12), recruited from the survey, to give researchers a closer understanding of the cleaning practices and the perceptions of cleanliness and hygiene, in relation to dust, of householders and the people who regularly clean homes as part of, and for, those households. The design ethnography observation and participant observation sessions (n = 25) took place in the same administrative districts; Adentan Municipal Assembly (n = 4), Ga East Municipal Assembly (n = 12), and La-Nkwantanang Madina Municipal Assembly (n = 9). Sites were targeted for purposive sampling on the basis of being in either a rural (n = 4) or urban (n = 8) setting and housing a low income (n = 4), low-middle income (n = 4), middle income (n = 2) or high income (n = 2).

### Microbiological dust sampling

#### Sample size

The sample size was based on the previous ethnographic studies^27^, with 12 households being recruited in this study for design ethnographic study and microbial analysis of dust samples.

#### Dust collection and processing

Households were provided with 50 ml sterile falcon tubes (Thermofisher Scientific, UK) and asked to sweep their rooms and collect the dust into the tubes provided. Once the dust samples were received at the Noguchi Memorial Institute for Medical Research (NMIMR), they were kept at 4 °C until processed. Each household dust sample was sieved using a sterile 100 µm sieve to remove large particles. The sieved samples were split into two and stored at 4 °C for further analysis which comprised bacterial culturing and antibiotic susceptibility testing. All microbial analyses described below were conducted at the NMIMR.

#### Bacterial culturing and selection

One gram of dust was suspended in 10 ml sterile Phosphate Buffered Saline (1X PBS) buffer and vortexed, serially diluted up to 10^–4^ and 100 µl of each suspension was spread on nutrient agar plates in triplicate followed by incubation at 37 °C for 24 h. After the incubation, up to 50 bacterial colonies were selected at random from each dust sample and tested for resistance on a selective media supplemented with Ampicillin (50 µg ml^−1^), Tetracycline (100 µg ml^−1^), Sulphamethoxazole (100 µg ml^−1^), Kanamycin (50 µg ml^−1^) and Chloramphenicol (75 µg ml^−1^). The plates were incubated overnight at 37 °C, and growth checked after 24–48 h. These represented antibiotics in common use in this region.

#### Bacterial identification using matrix-assisted laser desorption ionization time-of-flight mass spectrometry (MALDI-TOF)

A single bacterial colony was homogenized in 100 µl of 1 × PBS and then subjected to Matrix-Assisted Laser Desorption Ionization Time-of-Flight Mass Spectrometry (MALDI-TOF)^28,29^.

#### Determination of haemolytic activity

Single colonies were streaked on 5% sheep blood agar (Sigma Aldrich, UK) and observed for haemolysis after a 24-h incubation at 37 °C.

#### Multiple antibiotic susceptibility testing

The identified isolates were further assessed for their susceptibility against a panel of 28 antibiotics utilizing the disk diffusion method according to the Clinical and Laboratory Standards Institutes (CLSI 2009) on Mueller–Hinton agar (MHA: Sigma Aldrich, UK). Each isolate was re-suspended in 2 ml sterile 1 × PBS and vortexed. The turbidity of the suspension was adjusted to a 0.5 McFarland standard and 100 μL spread on to a Mueller–Hinton agar plate. After drying for 5 min, CLAIRO COMBI disk was aseptically placed on the medium and incubated at 35 °C for 18 h. If confluent growth was observed the diameter of the zone of complete inhibition was measured to the nearest millimeter and the results recorded. Antibiotic resistance was tested for Penicillin-G (10 IU ml^−1^), Amoxicillin (10 µg ml^−1^), Amoxicillin/clavulanic acid (20 µg ml^−1^ and 10 µg ml^−1^ respectively), Co-trimoxazole (25 µg ml^−1^), Cephalexin (30 µg ml^−1^), Cefotaxime (30 µg ml^−1^), Cefuroxime (30 µg ml^−1^), Erythromycin (15 µg ml^−1^), Chloramphenicol(30 µg ml^−1^), Cephalothin (30 µg ml^−1^), Ofloxacin (5 µg ml^−1^), Piperacillin (100 µg ml^−1^), Azithromycin (15 µg ml^−1^), Tetracycline (30 µg ml^−1^), Norfloxacin (10 µg ml^−1^), Aztreonam (30 µg ml^−1^), Cefotaxime (30 µg ml^−1^), Ceftriaxone (30 µg ml^−1^), Nalidixic (30 µg ml^−1^), Nitrofurantoin (300 µg ml^−1^), Cefuroxime (30 µg ml^−1^), Gentamicin (10 µg ml^−1^), Amikacin (30 µg ml^−1^), Cephalothin (30 µg ml^−1^), Ceftazidime (X µg ml^−1^), Cefixime (30 µg ml^−1^) and Cefdinir (5 µg ml^−1^).

The diameters obtained were analysed as resistant, intermediate or susceptible according to standard interpretative table by CLSI (2012). Multiple antibiotic resistance (MAR) index was calculated as follows:

$${\text{MAR }} = {\text{ a}}/{\text{b}}$$

where (a) is the number of antibiotics to which an isolate is resistant and (b) is the total number of antibiotics tested. MAR > 0.2 indicates that the isolates are from high-risk sources^30^.

MAR index of sample or sampling point (from which several isolates were taken) is estimated as follows:

$${\text{MAR Index S }} = {\text{ a}}/{\text{bc}}$$

where (a) is the aggregate antibiotic resistance score of all isolates from the sample or sampling point, (b) is the number of antibiotics tested, and (c) is the number of all isolates from the sample or sampling point. For the calculation, intermediate resistance was considered resistant.

### Co-design workshop

The co-design^31^ workshop (n = 7) took place at the NMIMR, University of Ghana, Accra, Ghana, with design researchers from the Lancaster University planning and directing the facilitation. Researchers from both institutions delivered the workshop. The workshop was intended to assist participants in drawing together the various strands of the investigation, in order that they might synthesise the findings while co-creating context-specific cleaning practices that could mitigate the impacts of AMR. The contexts, four economically segmented scenarios, were developed from the survey data, and identified different types of dwelling; a single-room compound house, a double-room compound house, a five-room apartment, and a ten-room semi-detached house.

To allow participants to actively explore the problem space and advance cleaning techniques in a representative setting we set out to create a doll’s house toolkit^32^ using 1:1 scale floorplans for each scenario to create four sites. To enable this, a large venue at local partner was requested for two days to prepare and run the workshop. The sites were partially developed from the scenarios however, additional insights were required to clarify the floorplans and the internal layouts of furniture and fittings, which were to be represented in the workshop by cardboard boxes. These smaller sites were presented roughly at a 1:1 scale. The two larger sites were slightly reduced in scale and limited to four rooms each to fit within the available space.

**Figure 1:**
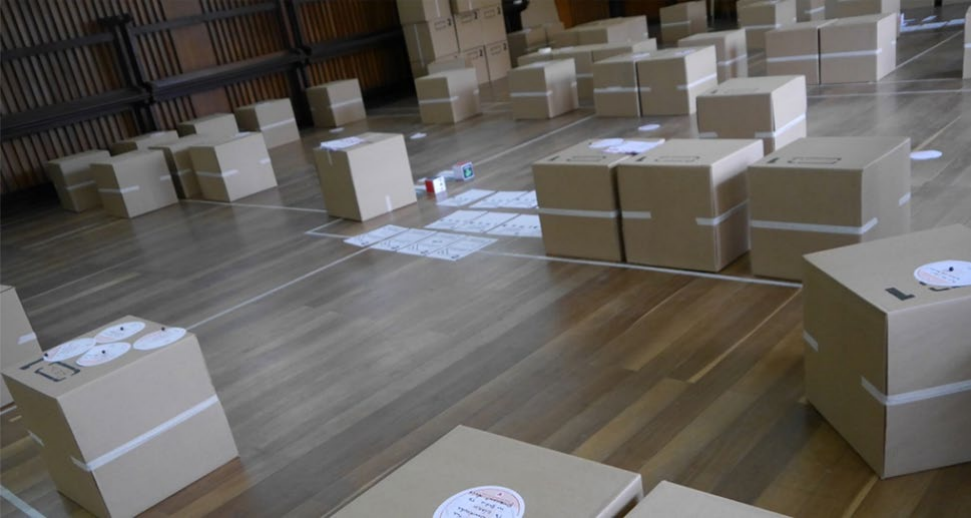
Simulated case study homes using floorplans and cardboard boxes.

Ten cardboard boxes were set up in each room and participants were asked at the outset of the workshop to decide what furniture and fittings would be present in each room (see Fig. 1). Participants wrote the item’s name on the box and placed it appropriately in the space. Participants were first asked to identify cleaning issues, which they did, while familiarising themselves with the scenario sites. Then they were to validate the internal layouts of the furniture and fittings in each site, making any changes necessary to better represent similar households in Accra, before clarifying the cleaning issues in the newly established layout.

**Figure 2:**
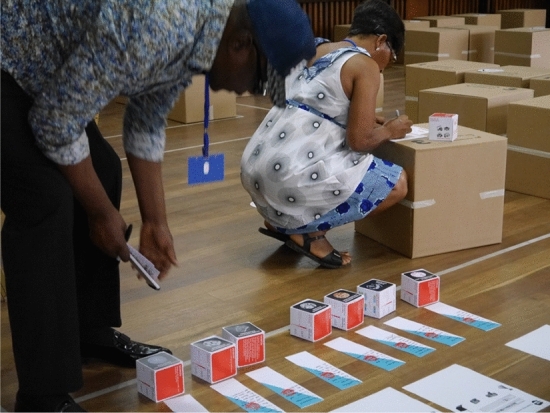
Participants engaging with workshop material. Large carton boxes representing furniture and small boxes representing bacteria.

**Figure 3:**
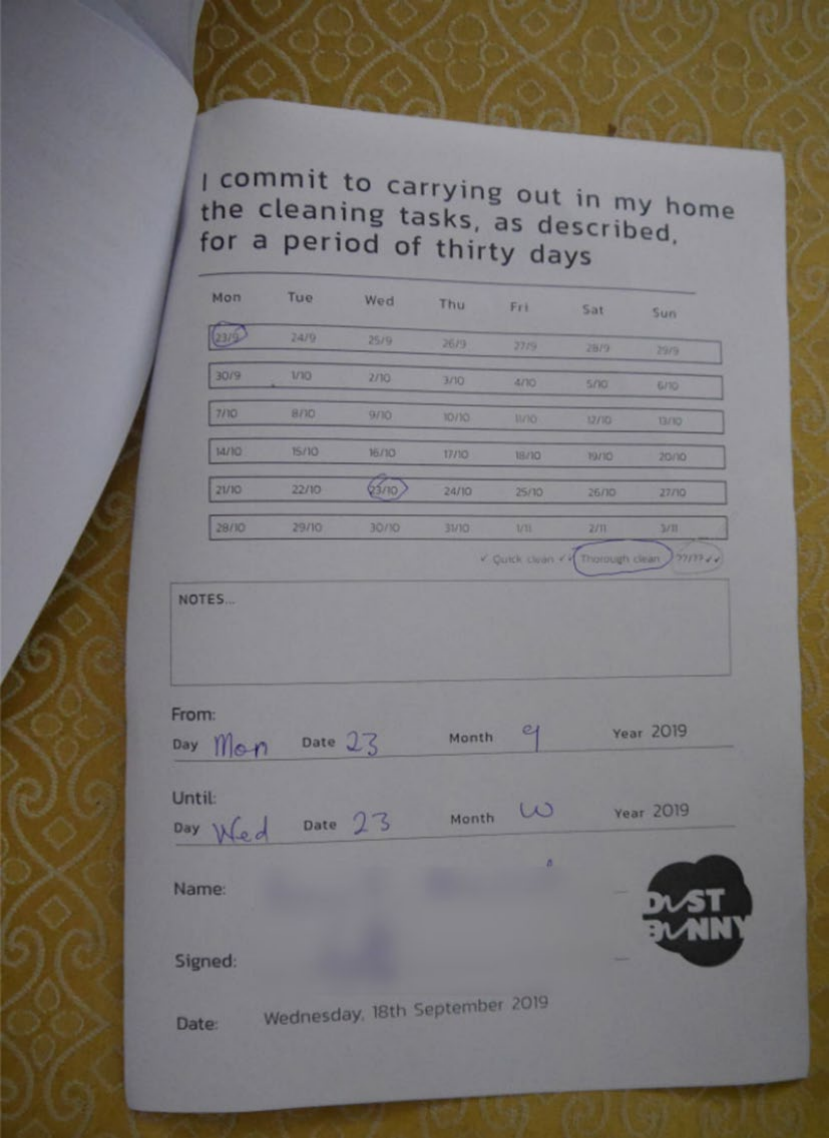
Individual cleaning agreement developed from the co-created cleaning practices.

They were also presented with small boxes which included pictures and information in layman’s terms of the different bacteria found through the microbiological study in their homes (see Fig. 2). By the end of the day, participants had co-created cleaning practices, reviewed them and selected appropriate ones to incorporate into their own cleaning regime and individual cleaning agreements (see Fig. 3).

### Cleaning post-intervention interviews

An intervention, a new regime of cleaning practices agreed through the codesign workshop and practiced for thirty days, was made in (n = 7) households and another round of microbiological sampling (n = 12) and analysis conducted to ascertain any impact on the domestic microbiome. A post-intervention interview was also conducted by going back to the households who participated in the co-design workshop. All these led to the development and dissemination of a cleaning education and information material.

### Qualitative data analysis

Qualitative data analysis was conducted for the design ethnography and the post intervention interviews. With regards to the design ethnography, working in pairs to triangulate the data, researchers at NMIMR produced household summaries by cross-checking the narrative accounts for the sessions from each household. This data was then passed to LU where the constant comparative method of analysis was used to generate the findings, with one researcher developing the categories and another researcher crosschecking their appropriateness, to ensure the analytical process produced credible themes^33^.

For the interviews, thematic analysis was employed^34^, where all data collected are involved in a process of identifying themes throughout coding, indexing, and categorizing towards drawing themes. More precisely, the code generation was done by looking at each paragraph of each chapter of the Design for Health kook and coding data, by writing notes through the use of sticky notes and electronic notes within the electronic version of the gook. After the data coding and collation, we started to look for overarching themes based on the area of interest and investigation.

### Ethics approval and consent to participate

The study has received ethics approval (Certified Protocol Number (CPN): 053/17-18) by the Institutional Review Board, Noguchi Memorial Institute for Medical Research, Ghana with Federal Wide Assurance Registration FWA 00001824. Study methods were performed in accordance with relevant national and international regulations and guidelines for conducting qualitative research. Informed consent was obtained from all subjects and/or their legal guardian(s). Also informed consent was obtained from all subjects for publication of identifying information/images in an online open-access publication.

## Results

### Survey data analysis

The survey was conducted by four female data collectors in the Accra Greater Region’s Adenta Municipal Assembly, Ga East Municipal Assembly, and the La Nkwantanang Municipal Assembly. It focused on respondents who had either held responsibility for directing household cleaning 99% (n = 250) or who had identified themselves as the primary cleaner of the household for a period over three years 90% (n = 227). The householders visited were across the socio-economic spectrum, the respondents (n = 251) ranged from 18 to 83 years old and were 86% (n = 215) female and 14% (n = 35) male, with one undeclared.

The survey engaged with an ethnically, religiously, and educationally diverse set of respondents across the socio-economic spectrum. The ethnic groupings were reported, as follows: Akan 41%, Ewe 27%, Ga/Dangme 12% (the indigenous ethnic grouping of the region), Mole-Dagbani 5%, and Other 15%. The religious affiliations were reported, as follows: Pentecostal or Charismatic 33%, Presbyterian 18%, Islam 13%, Catholic 8%, Methodist 5%, Protestant 6%, and Other Christian 17%. Educational attendance was broadly spread with respondents reporting attendance as follows; Primary 9%, Middle 9%, JSS/JHS 14%, Secondary 15%, SSS/SHS 18%, and Higher 35%.

The cleaning frequency indicated that a third of the household conduct cleaning daily (35%). Another third of participating households clean their homes either every two (11%) or three (20%) or four days (3%), and less than a third tend to clean once per week (25%). Only a handful of households conduct cleaning every two weeks (3%) or once per month (3%).

Cloth was reportedly used to clean dust among the households interviewed (87.9%) over all (96% among the LIG, 90% among the LMIG, 87% among the MIG and 78% among the HIG. Cloth is used to clean at least once in a week across most socioeconomic groups with 3% who clean once every 2 weeks or every month. Respondents used mainly bleach solution (63%) to clean the cloths that they use for cleaning (77% among the LIG, 66% among the LMIG, 64% among the MIG and 39% among the HIG) however, 61% of the High-income households use non-bleach detergent for cleaning the cloth.

The use of sponge to clean dust was common across the socioeconomic groups (69.2% among the LIG, 59% among the LMIG, 65% among the MIG and 75% among the HIG, giving the over all of 65%. Bleach solution (62% among the LIG, 52% among the LMIG, 54% among the MIG and 63% among the HIG) was commonly used to clean the sponge used for cleaning. Others include Non-bleach detergent (16% among the LIG, 33% among the LMIG, 34% among the MIG and 33% among the HIG) and Water (21% among the LIG, 12% among the LMIG, 8% among the MIG and 3% among the HIG).

Dustpan and Brush was one of the most popular cleaning tools among respondents (98%) among all groups (92% among the LIG, 97% among the LMIG, 100% among the MIG and 100% among the HIG). The Dustpan and Brush are mainly used on daily basis (91% among the LIG, 68% among the LMIG, 75% among the MIG and 69% among the HIG).

Sweeping Brush was not so popular among the LIG (18%) but highly popular among the rest of the socioeconomic groups interviewed (LMIG 62%, MIG 73% and HIG 91%). The Sweeping Brush is used mainly on daily basis to clean among those who use it (92%), thus 100% among the LIG, 93% among the LMIG, 93% among the MIG and 91% among the HIG). The Sweeping Brush is cleaned mainly on daily basis using water among the LIG, whereas the other socio-economic groups use water, Bleach solution and non-Bleach detergents to clean it.

### Rapid ethnography data analysis

Within-method methodological triangulation^35^ was used through the combination of observation and participant observation methods. This was further supported by researcher triangulation with each site was visited by two researchers; one to conduct the observation session, and one to conduct the participant observation session.

During the review of the data fifteen recurrent themes emerged.

1. Religion, superstition and traditional practice
2. Individual creed over religious practice
3. Social judgement
4. Taught knowledge and situated practice
5. Negotiated practice
6. Cleaning styles are often tacit practices
7. Repetition
8. Different brooms for different rooms
9. Walking dirt in and sweeping dirt out
10. New and old or new for old
11. Chemical storage and safety practices, strong smells and mitigations
12. Dusting, sweeping, scrubbing and mopping
13. Tools
14. Waste disposal
15. Seasonal Variation

#### Religion, superstition and traditional practice

We observed little connection between religious practice and cleaning practice across faith traditions, but within the ethnographic accounts there were limited signs that superstitions influence current practice.

We found only one link with cleaning practices and any tribal or ethnic traditions; however, the same omission of activity was carried out without ethnic dimension being acknowledged by other participants.

#### Individual creed over religious practice

Though people may practice their religious faith diligently it does not necessarily have a specific impact on their cleaning practices. Rather, cleanliness may be understood as a part of a person’s individual creed and is not attributable to a particular religious dogma.

#### Social judgement

In a similar fashion, as one researcher noted, one participant “insults people when she visits their bathroom and [sic] is unclean, so she is always careful with hers, so that no one insults her when [sic] visited.” From the study we understand that this judgemental characteristic is commonplace in Ghanaian culture, as it is to varying degrees the world over, and so we need to consider how it relates to the challenges of recruitment for research and any future intervention strategies. The social judgement of others may be a powerful motivator for some cleaning practices, especially those that are visible to others, but what does it mean for people to judge and to be judged in this context? Deeper understanding may be found by adopting social psychology perspectives to explore these questions as they relate to behaviour change theory.

#### Taught knowledge and situated practice

The relationship between individual problem solving and taught practices is difficult to disentangle. General practices are taught, by family members (primarily female, but not exclusively) at the household and in school settings, however situated problems arise that fall outside of the taught knowledge and require the individual to develop particular cleaning solutions, for example a new household may require new skills as the dwelling has different material properties. To encapsulate we found that what you clean is strongly linked with how you clean.

#### Negotiated practice

As a site-specific practice cleaning has to adjust to countless factors. It is influenced by the material properties of the dwelling, a combination of an individual's taught and tacit knowledge, and is negotiated around the presence of other people, and day-to-day health, safety and storage concerns visible within peoples’ activities in the household.

#### Cleaning styles are often tacit practices

Specific cleaning actions, such as mopping styles, may have no specific rationale or precedent that can be articulated explicitly^36^ by participants, there is however an implicit understanding or feeling about what constitutes appropriate cleaning practice. For example, “I asked why she mops in two patterns/ways and where she learnt them from? She said no one, but she feels that’s the right way.”

#### Repetition

As may be inferred from the above quotation, the cleaning styles often make use of actions that repeatedly cover the same ground to ensure a clean floor. To varying degrees repetition is also an explicit part of some cleaning practices, such as with mopping. It may be that the mopping style has repetition built into a single action, such as the zig-zag pattern often employed by participants, or it may extend to the entire room being mopped for a second time, sometimes as a punishment from a third-party to ensure their standards are met, and sometimes as a way of meeting a person’s own standards.

#### Different brooms for different rooms

Some participants make a direct link between sickness and germs in the household, particularly when it comes to floors and children. They seek to protect their health by establishing practices to control the potential transmission of germs throughout the household, such as using different brooms to sweep different rooms. However, this may also be taken to an extreme with some indication of performative cleaning practices in some households, a practice which links to the issue of social judgement.

#### Walking dirt in and sweeping dirt out

Transmission of dirt from outside the home to its interior is reduced in two ways, one strategy is to wear indoor shoes, another strategy is to go barefoot during cleaning. Bare feet allow for the sense of touch to be employed, during cleaning the soles of the feet are themselves cleaned to ensure that dirt is detected.

Dirt and dust are swept from inside towards the outside of the dwelling. This simple phenomenon was repeatedly observed by participants, as one noted, “[…] when you go outside you bring dust or dirt inside the room so it advisable to sweep from inside to outside and since I was born, I always see my mum and other people sweep from inside to outside.” Though this practice links to superstition the individual act is not necessarily conducted with this mind.

#### New and old or new for old

Most participants use both traditional and modern tools, with market introduction supported by commercial products that provide “self-explanatory” learning materials to help people take on new cleaning methods. However, the adoption of new cleaning techniques is considered by some participants as being in part a result of the acceptance of modern ways and the rejection of traditional tools and techniques. While others proclaim a distinct preference for both traditional tools, and local bleach solutions.

#### Chemical storage and safety practices, strong smells and mitigations

There are safety concerns regarding young children coming in to contact with cleaning products. These are addressed by various storage strategies to mitigate potential harm. However, storage priorities are predominantly related to proximity of use.

We observed a high level of the use of unbranded local cleaning products. A wide range of branded products were also in use.

To mitigate the effect of strong-smelling chemicals, various strategies were employed. In one striking case a makeshift facemask was created using a participants mayafi, a large cloth that is a traditional part of Muslim women’s dress. More regularly however, participants varied the duration of application times before rinsing away with water. Masking scents were also employed to reduce the impact of the chemicals.

With high levels of use of unbranded products there is a possible issue with variability of product contents and with a potential uncertainty of provenance.

#### Dusting, sweeping, scrubbing and mopping

With some slight variations generally shared practices exist within the most common four cleaning activities like dusting, sweeping, scrubbing, and mopping.

- Dusting is conducted using a range of cloths, rags, and sponges, both with the addition of water, or disinfectant, and without. The subjects of dry dusting are raised surfaces and objects of various value, including ornaments and books to computers, sound systems and televisions. While subjects of wet dusting include difficult to access areas, furnishings and accessories such as under microwave ovens, overhead fans and louver blades.
- Sweeping is most commonly practiced with a traditional broom, though long handled brooms are also in use. Two techniques for using the traditional brooms are highlighted, dependent on the nature of the sweeping task, with the broom held either sideways or upright. While no one technique of sweeping with a long-handled brush is apparent across the study. When sweeping is conducted the same general pattern of activity can be observed. It begins with sweeping from the sleeping area, through to the hall, gathering any debris in the centre of rooms, collecting detritus in a dustpan and disposal.
- Scrubbing with handheld hard bristled brushes and brooms in concert with a range of bleaches, detergents and antiseptics is regularly carried out in all instances. The bathroom and the toilet are the focus of most scrubbing techniques, particular attention is commonly paid to ‘the corners, the drain and the joints of the tiles’ in these settings. A variety of practices were witnessed in regard to the length of application, and the washing away, of disinfectants, and the management of their associated chemical scents.
- While mopping techniques included moving the mop head from left to right and vice versa and the figure of eight, the most commonly described technique for mopping is a zig-zag action, performed moving backwards. This is considered the most effective technique by the majority of participants at removing dust, dirt and grime from the floors.

#### Tools

The careful care of cleaning implements was observed in most sites. One participant described the common practice, thusly, “I always use washing powder and water to clean the tools and dry them in the sun. I always wash the tools I use, dry them and pack them neatly after each cleaning.”

#### Waste disposal

No clear picture arose regarding waste disposal. Sanitation services are mentioned only in high income areas. Waste from dust pans is commonly sided to an external bin, in some settings however it is disposed of into the road or outside the compound. The accounts did not extend beyond the disposal in most cases. There was one recorded instance of burning toilet wastepaper, the practice is reportedly not uncommon. It should be noted that if the burning of toilet waste is done poorly faecal matter may return as airborne particulate matter.

#### Seasonal variation

Seasonal variations in cleaning practices were reported. The increased wind and dust in the dry season create more cleaning work.

### Microbiological data analysis

We employed microbiological analysis to inform intervention design and give the risk from AMR some context. Bacteria from each sample, that showed resistance to one or more antibiotics were retained, and some were identified to species level and then classified as an obligate pathogen (always causes disease), an opportunist pathogen (can cause disease when the host is compromised) and a non-pathogen (no history of causing disease).

Sampling regime comprised collecting dust samples from 12 houses which represented houses from high (HIG; n = 2), Middle (MIG; n = 2), Low-middle (LMIG; n = 4) and low income (LIG; n = 4) groups in North Legon, Adenta, Madina, and Abokobi, respectively and the numbers of bacterial isolates obtained and purified for further analysis is shown in Table 1.

**Table 1:** House dust samples location in Accra, Ghana, socio-economic grouping and sources (B- Bedroom, H-Hall, K-Kitchen and W-Windows; High Income Group (HIG), Middle Income Group (MIG), Low-middle Income Group (LMIG) and Low Income Group (LIG)).

Sample number	Socio-economic category	Community location	Area of house sampled	Number of isolates
001	LIG	Abokobi	B, H, K	62
002	LIG	Abokobi	B, H, K, W	25
003	LIG	Abokobi	B, H, K, W	43
004	MIG	Adenta	B, H, K	33
005	LMIG	Madina	B, H, K	44
006	LIG	Abokobi	B, H, K, W	43
007	LMIG	Madina	B, H, K	36
008	LMIG	Madina	B, H, K, W	44
009	LMIG	Madina	B, H, K	38
010	MIG	Adenta	B, H, K, W	35
011	HIG	North Legon	B, H, K, W	42
012	HIG	North Legon	B, H, K, W	36

#### Bacterial identification

From the dust samples, 481 isolates were examined and 36.6% (n = 176) were found to carry at least one resistance to the initial panel of antibiotics tested. Of the resistant isolates, 119 were subjected to MALDI-TOF with 86 returning identifications. The identity (and relative numbers from all the samples) of the 11 bacterial species comprised *Acinetobacter baumannii* (2.5%), *Acinetobacter radioresistens* (4.2%), A*cinetobacter* sp (2.5%), *Arthrobacter mysorens* (0.8%), *Bacillus cereus* (51.3%), *Bacillus thuringiensis* (0.8%), *Enterobacter cloacae* (4.2%), *Macrococcus caseolyticus* (0.8%), *Pseudomonas stutzeri* (0.8%), *Staphylococcus arlettae* (0.8%), *Staphylococcus saprophyticus* (2.5%). Some isolates (28.6%) could not be identified within the limitations of the methods used. *Bacillus cereus* dominated the diversity of resistant isolates (52%) in all locations**.** In addition to *Bacillus cereus*, LMIG comprised 8 different species namely *Acinetobactor radioresistens, Acinetobactor sp., Arthrobacter mysorens, Leclercia adecarboxylata, Pseudomonas stutzeri, Staphylococcus arlettae and Staphylococcus saprophyticus.* The LIG sample comprised *Enterobacter cloacae, Acinetobactor radioresistens, Acintetobacter baumannii, Exiguobacterium auranticum* and *Macrococcus caseolyticus.* HIC revealed one additional species*, Bacillus thuringiensis.* HMIG comprised only *Bacillus cereus*.

#### Resistance profile of cultured isolates

Of the 176 isolates that were found to carry at least one resistance to the initial panel of antibiotics tested (ampicillin, tetracycline, sulphamethoxazole, kanamycin and chloramphenicol), the most common resistance was to sulfamethoxazole (75%) with all *Bacillus cereus* being resistant followed by tetracycline (16%), chloramphenicol (7%) and ampicillin (2%). The resistance varied between the socio-economic groupings with LIG showing the highest proportion (56%), and the LMIG, MIG and HIG, all showing lower resistances (30.8%, 26.6% and 22.1% respectively). Within the 176 isolates identified as resistance to one antibiotic 7% showed resistance to 2 antibiotics and 1% showing resistance to 3 antibiotics. One unidentified isolate from LMIC was resistant to all 5 antibiotics tested including kanamycin.

#### Community level antibiotic resistance

Table 2 below shows the bacteria strains resistant to either ampicillin, tetracycline, sulphamethoxazole or chloramphenicol, identified in each socio-economic group. Overall, 36.6% (176/481) isolates showed at least one resistance with 56.2% (91/176) of resistant isolates being obtained from the LMIG. The majority were resistant to sulfamethoxazole. In the LIG, HIG and MIG, although sulfamethoxazole resistance predominated, the resistant bacteria were less prevalent at 26.6% (46/173), 22.1% (15/68) and 30.8% (24/78) respectively. A higher number of multiple resistance isolates was observed in LIG and LMIG class followed by MIG. Only sulfamethoxazole resistance was observed in upper class. Samples from all classes were dominated by *Bacillus cereus.* Within these strains, 33 were tested for haemolytic activity. ß- haemolytic activity (complete lysis of blood cells) was observed in 12 (36%) isolates, including species of *Bacillus cereus, Enterobacter cloacae, Exiguobacterium auranticum, Pseudomonas stutzeri* and *Staphylococcus saprophyticus*; 21 (61%) showed **γ** -haemolytic activity (no activity). No isolates showed **α** -hemolytic activity. ß-hemolytic activity was observed in all communities but was higher in LIG.

**Table 2:**
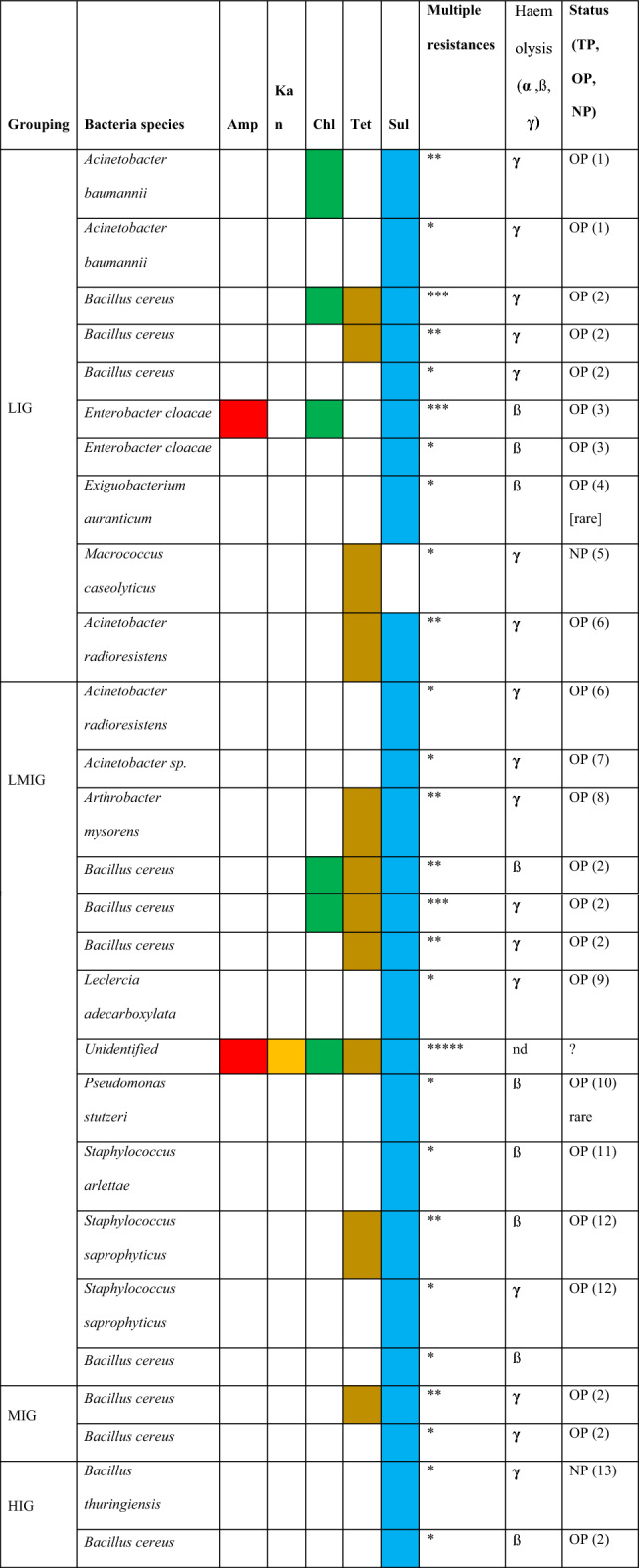
Examples of resistance profile combinations observed within different bacteria species and socio-economic grouping.

Resistant bacteria identified. Star ratings denotes the number of resistances observed in the species; *1 resistance; **1–2 resistances; ***1–3 resistances observed in isolates. *TP* true or obligate pathogen to humans, *OP* opportunist pathogen to humans, *NP* non pathogen to humans.

*Amp* ampicillin, *Kan* kanamycin, *Chlo* chloramphenicol, *Tet* tetracycline, *Sul* sulphamethoxazole.

##### Antibiotic susceptibility testing

**Figure 4:**
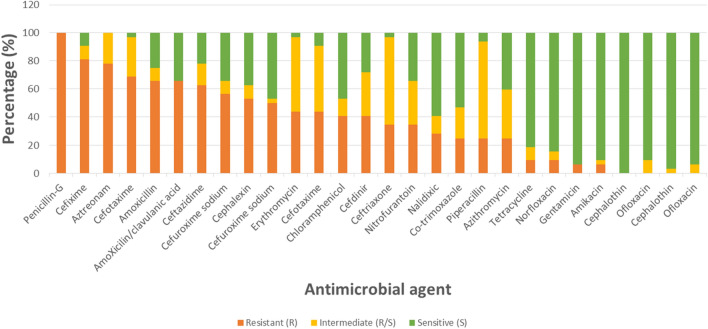
Susceptibility outcome to different antimicrobial agent.

The thirty three resistant isolates tested for haemolytic activity were subjected to further susceptibility testing against a panel of 28 antimicrobial agents. All isolates were resistant to penicillin, and least resistant to ofloxacin (Fig. 4).

**Figure 5:**
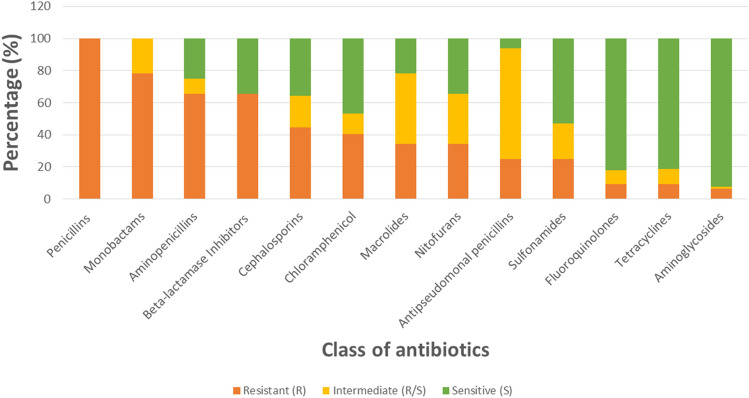
Susceptibility to different classes of antibiotic.

When the antimicrobial agents are grouped into various antibiotics classes, the isolates were more resistant to the penicillins and least resistant to the aminoglycosides (Fig. 5). In terms of socio-economic grouping the level of resistance was not significant between the various classes (p > 0.05).

With respect to the presence of isolates with multiple resistances, the MAR S index for the different species ranged from 0.4 to 0.7 (Table 3) indicating that a greater proportion of isolates carry multiple resistances. When estimated for the sample points (according to socioeconomic grouping), the MAR index ranged from 0.3 to 0.6 (Table 4), indicating all areas contained multiplely resistant isolates with the LIG (0.6), LMIGg (0.5) and MIG (0.6) being at higher risks compared to the HIG (0.3).

**Table 3:** Number of antibiotics to which identified species of bacteria are resistant or sensitive.

Species	MAR index
*Acinetobacter baumanii*	0.7
*Acinetobacter radiorenstens*	0.6
*Acinetobacter *sp.	0.6
*Arthrobacter mysorens*	0.5
*Bacillus cereus*	0.6
*Enterobacter cloacae*	0.6
*Exiguobacterium auranticum*	0.7
*Lecteria adecarxylata*	0.7
*Macrococcus caseolyticus*	0.4
*Pseudomonas stutzeri*	0.7
*Staphylococcus arlettae*	0.4
*Staphylococcus saprophyticus*	0.7

All isolates have multiple resistance.

**Table 4:** MAR S index according to socio-economic grouping.

Socio-economic classification	MAR S index
LIG	0.6
LMIG	0.5
MIG	0.6
HIG	0.3

### Co-design workshop and cleaning intervention data analysis

The process, findings and insights were anonymised and visualised to provide the material for co-designing cleaning practices, that were effective, easy to communicate and specific to the communities in question. Specifically, four scenarios were generated from an economic segmentation of the survey data, 50 ethnographic insights were ‘presented’ and descriptions of 12 bacteria, that represented the 125 identified species found in the dust samples, were presented to the participants in a co-design workshop.

Respondents described their participation in the 30-day cleaning contract that they signed with the project in varied ways. They generally described it as worthwhile and a period of learning and reflection. The most important element though was evidence of behaviour change. The workshop and the cleaning contract following this, enabled participants to re-evaluate their current cleaning practices and make significant changes, as seen from the quotes below.

“At first, I used to sweep without raising items like chairs to sweep under them but after the workshop it was put in my contract to raise stuffs and clean under them to remove dust […] Within these 30 days, my cleaning has been different, it has been more and it has been a very worthwhile experience” (Female, Lower middle income/working class community).

“During the thirty days cleaning contract period, I purchased a new mop towels and disinfectant, which I used for mopping the floor for a better result. My cleaning during this time was more intensive as I dusted every item in my rooms. I also used different tools in cleaning the toilets, the rooms and the kitchen” (Male, Middle income community).

Although, not intended to do so, the process of asking each participant to complete the cleaning contract for their own household revealed cleaning practice nuances, which were missed or not collected during the design ethnography. For example, looking at the quotes above and below, one can see that past cleaning practices were not always thorough, did not make use of detergents, were not done consistently and reused as much as possible the same cleaning tools for all rooms. Furthermore, it is interesting to see that findings presented during the workshop (i.e., bacteria found underneath furniture during the microbiological dust sampling) influenced new cleaning behaviours as participants took these on board in the development of their new cleaning interventions, outlined in the cleaning contracts.

“My 30 days cleaning exercise was great, compared to my normal cleaning practices., where I clean only in the morning, after the workshop, I now clean twice in a day. […] I now combine detergents like bine or Parazone and washing powder for the cleaning “(Male, High income community).

“Over the last thirty days, I have been cleaning my room every day. Before, I could go for five days without cleaning, thinking I am alone and therefore, there is no need to clean the rooms every day because it's not dirty” (Female, Low income/poor community).

In terms of compliance with the cleaning contract, participants overall followed their contract ‘religiously’, though in most cases they had to do things differently. For some, they just wanted to see if cleaning according to what was written in the contract would make their homes neater.

“I used to do the cleaning weekly but because of the contract I did it daily for 29 days, missing only one day out of the contract period of 30 days, I really wanted to see if there will be any change in my rooms, and I can say that there was, the rooms look cleaner than before” (Female, lower middle income/Low-middle income community).

For others it was because they learnt from the workshop that there are bacteria in their homes, so they wanted to do whatever possible to reduce or eliminate these.

“I did what I said I will do, that is cleaning on a daily basis because there is lot of dust in my area and I now know from the workshop that the dust carries bacteria into our homes, some of which can cause infections. I also added disinfectant to the water I used for cleaning floors and other surfaces in my home” (Male, Middle income community).

“I did all what I said I would do in the contract […] I am sure that the dust from room will record low bacteria” (Male, High income community).

Moreover, the post-intervention interviews provided more details on the amount of work and level of commitment required by workshop participants during the 30-day cleaning contract period. As one can see from the quote below, adopting the new cleaning practices, following the findings presented at the workshop, was time consuming. Nevertheless, as participants could see the impact of their effort and understood the why (bacteria present at the household level that can cause disease) before the what (i.e. lifting furniture, using different cleaning tools for different rooms, etc.) it changed the way they cleaned (the how) and enhanced their resolve in continuing with the new cleaning practices post-intervention.

“It requires commitment to do it because at first, I only sweep under my bed up to where I can see or reach but now (during the contract period) I have to push my bed when cleaning and then push it back to its normal position after cleaning. […] lift up things on the floor when cleaning to clean under them […] it was time consuming, but the end result is good. I managed to clean according to the contract, twice everyday but I couldn’t complete all the 30 days, I did it for 28 days because I have to be out of the home for a few days. […] I have resolved to continue practicing what I have signed to do even after the project”. (Female, Lowe-middle income/working class community).

We sought to find out from participants, who took part in the observational study and participated in the co-design workshop, whether the personalised interventions developed influenced their cleaning practices. We discovered that they were influenced by the project in one way or the other as represented in the narratives below. More precisely, we documented new cleaning practices adopted. Most of these practices refer to (a) a more structured approach to cleaning at the household level; (b) increasing the cleaning frequency; (c) use of cleaning detergents; and (d) use of different cleaning material for different surfaces and rooms.

“The project, especially the workshop has influenced my cleaning. At first, we clean the sink before cleaning the bath but now, we clean the bath, then the sink, also wash and dry the brush before use again” (Female, High income community).

“The project has influenced me […] I used to clean ones a week, now I do it twice or sometimes three times in a week… “(Female, Lower income/working class community).

“I learnt so much from the project to improve on my cleaning. At first, I used only washing powder but now I use detergent to wash and clean. I now use different brash and bucket to clean the toilet area put detergent into the closet after use to protect the next user infection” (Female, Middle income community).

“[…] before the project, I used to mop using the same mop for all the rooms (living, sleeping, kitchen, toilet and porch) but I realized from the project that it is not a good way of cleaning” (Female, Low income/poor community).

## Discussion

### Current cleaning practices and AMR: household perceptions

The findings (from the survey) reveal that although most participating households (85%) recognise dust in their rooms as a cause for concern, a lesser number (74%), believe there is a connection between dust and germs. Over half (72%) of those recognised germs found in dust being associated with cold and associated symptoms such cough, catarrh, headaches. Only a handful associate dust with other health conditions, such as asthma (7%), cholera (5%) and Tuberculosis (3%). These are similar to findings from South Africa, where only 8% of low-income households believed that germs are aetiological agents in disease, while 40% associated dirt as an aetiological agent^37^. Additionally, a significant number of respondents (64%) consider the presence of dust at the household level to be normal. This was an issue that was unpacked further during the design ethnography. Although across all income groups the cleaning frequency is generally high (2–3 days per week) to very high (daily or four times per week), the cleaning practices in relation to the use of disinfectants varies. The majority (84%) of LIG use only water to clean hand brushes, in contrast to other income groups who use bleach solutions too. Overall, only a fifth across all income groups use bleach solution to clean the sponge.

The findings from the design ethnography touch on the underpinning beliefs and contemporary practices of a small number of households in Accra across the socio-economic spectrum. We note that each persons’ individual creed plays a significant role in the performance of cleaning practices, while the role of religion, superstition and tradition in cleaning practices is limited. This contrasts with^38^ approach of employing religion as a tool to encourage better waste Management in Ghana. The social judgement of others is a strong motivator for action and a mixture of school and familial taught knowledge and individual situated problem solving assure the utility of cleaning techniques. This contrasts with countries where hygiene behaviours are endemic, as social norms may not be as important as expected because people just grow up knowing they are supposed to do them^39^.

The site-specific and repetitive nature of cleaning activities mean that they are shaped by many other factors from a dwelling’s material properties to cohabitant’s preferences and to personal health, they are also often tacit in nature. Interestingly, Aunger’s study^39^ -a survey study with 1000 sample size in UK, USA, Canada, France, Germany, Australia, South Africa, Malaysia, Brazil, Middle East, China, and India- revealed some similarities. The results on household cleaning suggest that cleaning was seen as part of the daily or weekly routine, in response to a person’s general sense of a need for order in the household. Cleaners also paid attention to social norms to see whether other people in their network are doing the same. However, unlike our findings, they observed that, unlike in our study, this was not due to social pressure, i.e., from friends, but simply noticing that it is a common activity in their social circle^39^. Although difficult to extract the data for South Africa, which was the only African country in the study, it is interesting to note the principal difference in the social norms of household cleaning, between their and our study.

Both modern and traditional tools are in usage and there is a significant degree of care taken in their maintenance. They are used in conjunction with locally produced cleaning agents and imported European cleaning products. Use of local unbranded bleach solution products with very strong chemicals was evident. Some were so string that householders had to use masks or tried to mask the scent. This raises questions on the product contents reliability and effectiveness. While overall the use of both modern and traditional tools, and the locally produced and imported cleaning products elicited little controversy among our participants, some tensions were expressed with regard to the perceived erasure of Ghanaian practices.

Participants actively sought to protect their health by establishing practices to control the potential transmission of germs throughout the household including the establishment of boundaries for tools and developing strategies to control the transmission of dirt through the household. The dusting, sweeping, scrubbing, and mopping of households were conducted in a broadly similar manner in support of these practices. Also, the use of different tools, brooms for different rooms was an established practice in some households.

Reported findings point toward the need for further investigation of the impacts of both household waste disposal and seasonal change on the cleaning practices employed in Ghanaian homes. There was very limited sanitary and waste disposal for LIG, LMIG. Waste disposal tended to take place outside the home onto the street. In Ghana, most of the principal streets are filled with heaps of trash and the drains are choked with uncollected garbage causing flooding^38^. However, this is not a Ghanaian problem per se, as managing waste disposal in developing countries is one of the costliest services as it takes up to 1% of the gross national product and typically absorbs between 20 and 40% of municipal revenues^40^.

### Bacterial and AMR analysis

In dust and outdoor air, microorganisms are ubiquitous and diverse, and they can represent a large fraction of aerosolized particles^41^. Exposure to some of these microorganisms can have detrimental effects on human, animal, and plant health. Diversity of dust and associated organisms can be driven by different source environments can vary across seasons, land-use types, and vegetation types and local conditions and climate change^41,42^. Furthermore, AMR associated with bacteria in dust has been described but mainly in industrial facilities such as abattoirs and farming establishments^43^. Reference^44^ described the resistome (all the antibiotic resistance genes in communities of both pathogenic and non-pathogenic bacteria;^45^ in household dust and showed the significance of anthropogenic impacts and house location in structuring bacterial and fungal communities inside homes, respectively, and suggest that household dust is an overlooked reservoir for antibiotic resistance^44^. Reference^46^ carried out a systematic review of antimicrobial resistance in Africa. They concluded that recent AMR data is not available for more than 40% of the countries; that the level of resistance to commonly prescribed antibiotics was significant. Third, the quality of microbiological data is of serious concern.

Focussing on households in Accra Ghana, we examined AMR in culturable bacteria. Accra lies on the coast in southern Ghana, a narrow ecological strip of Coastal Savanna. This area has low precipitation and is characterized by scrubs and grasses. The coastal sandy soils consist of pale-yellow sands often without a surface layer of humus or organic matter. The Coastal Savannah ochrosols are similar to soils found in the Interior Wooded Savanna except that they are not as acid^47^. It is these that dominate the dust entering households that combine with other anthropogenic particles such as skin, hair and fur. The dust particles comprise part of the total house microbiome and therefore contribute to the resistome. Assuming cleaning involves all surfaces, removal of the dust from the study houses still removes bacteria which have an array of resistances reflecting those antibiotics used in the community as well as, in these cases, opportunist pathogens that threaten those who are already compromised with conditions that reduces immunity^48^. Multiple resistances prevail that reflect community antibiotic use and would further complicate treatment. We have shown, here, that householders are ‘at risk’ from infections whose treatment may be comprised if the same common antibiotics used in the community are part of their therapy^49^. Therefore, the need for adequate cleaning strategies coupled with appropriate prescribing practices is a necessary to limit or reduce the household resistome which address some of the recommendations of^50^ for global management of AMR.

Most studies on AMR have been restricted mostly to hospital settings and health workers. This study set out to demonstrate the potential for dust to carry AMR bacteria into homes. The results of the study indicate the presence of a great variety of MAR bacteria in the home environment, and this has the opportunity to compromise the effectiveness of antibacterial treatment and control. In hospital settings in Ghana, AMR in bacterial infections are related to *Escherichia coli*, *Pseudomonas* spp., *Staphylococcus aureus*, *Streptococcus* spp., and *Salmonella enterica* serovar Typhi^51^. In this study we observed multiple resistance in a range of opportunistic pathogenic bacteria isolated from dust. While most of the bacteria isolate were non-hemolytic, twelve bacteria isolates showed complete hemolysis of red blood cell. Bacteria hemolysis is the process of breakdown of red blood cells before the end of their life span^52^, indicating pathogenic potential of bacterial species. As such, *B. cereus, E. cloaca, C. casei, E. auranticum, P. stutzeri* and *S. saprophyticus* represent opportunistic bacteria species identified in the dust, with the potential to cause serious disease in humans. Furthermore, as a worldwide public health phenomenon, the increase of bacterial resistance to various antibiotics threatens their chemotherapeutic application^50^. In this study, 100% resistance was observed against the penicillins and more than 60% resistance to the beta-lactamase antibiotics.

Finally, all resistant bacteria identified in this study had a MAR index > 0.2 (0.4–0.7). The MAR index indirectly concludes that all the strains originate from an environment where antibiotics are often used^53^. As such all the resistant isolates are from high-risk sources of antibiotic resistance, pointing to the fact that antibiotic resistance is not limited to hospital acquired pathogens but can also be from community acquired pathogens. However, the link with hospital acquired resistance cannot be completely overruled considering the lack of proper drainage systems and the frequent occurrence of flooding during the rainy season in many parts of Accra. The MAR index (0.3–0.6) according to socio-economic grouping indicated that all the areas samples were highly contaminated, with the upper-class areas being the least contaminated. The presence of high antimicrobial resistance is of concern especially in developing countries with poor health and sewage infrastructures, coupled with high burden of infectious disease such as HIV/AIDS and tuberculosis which predisposes patients to infection by opportunistic pathogens. Further studies on the importance of these findings need to be undertaken, especially in children and immunocompromised individuals. The high prevalence of multidrug resistance observed in this study indicate the need for antibiotics surveillance program, not only in hospital settings but also in the home environment.

### Setting up the intervention and how design and microbiology allowed this

The setting up of the intervention through the co-design workshop, provided several benefits.

First, creating a forum where data collected could be shared with the participating households increased community engagement and dissemination. This required some effort translating the data from the design ethnography and microbiological study and visualising it in ways that could be used for sense making by the research participants. Typically, most of the research in developing contexts do not share the findings with research participants, creating the notion of ‘mosquito researchers’^54^; or do not share them in a format that is easily understood and usable by the research beneficiaries^55^.

**Figure 6:**
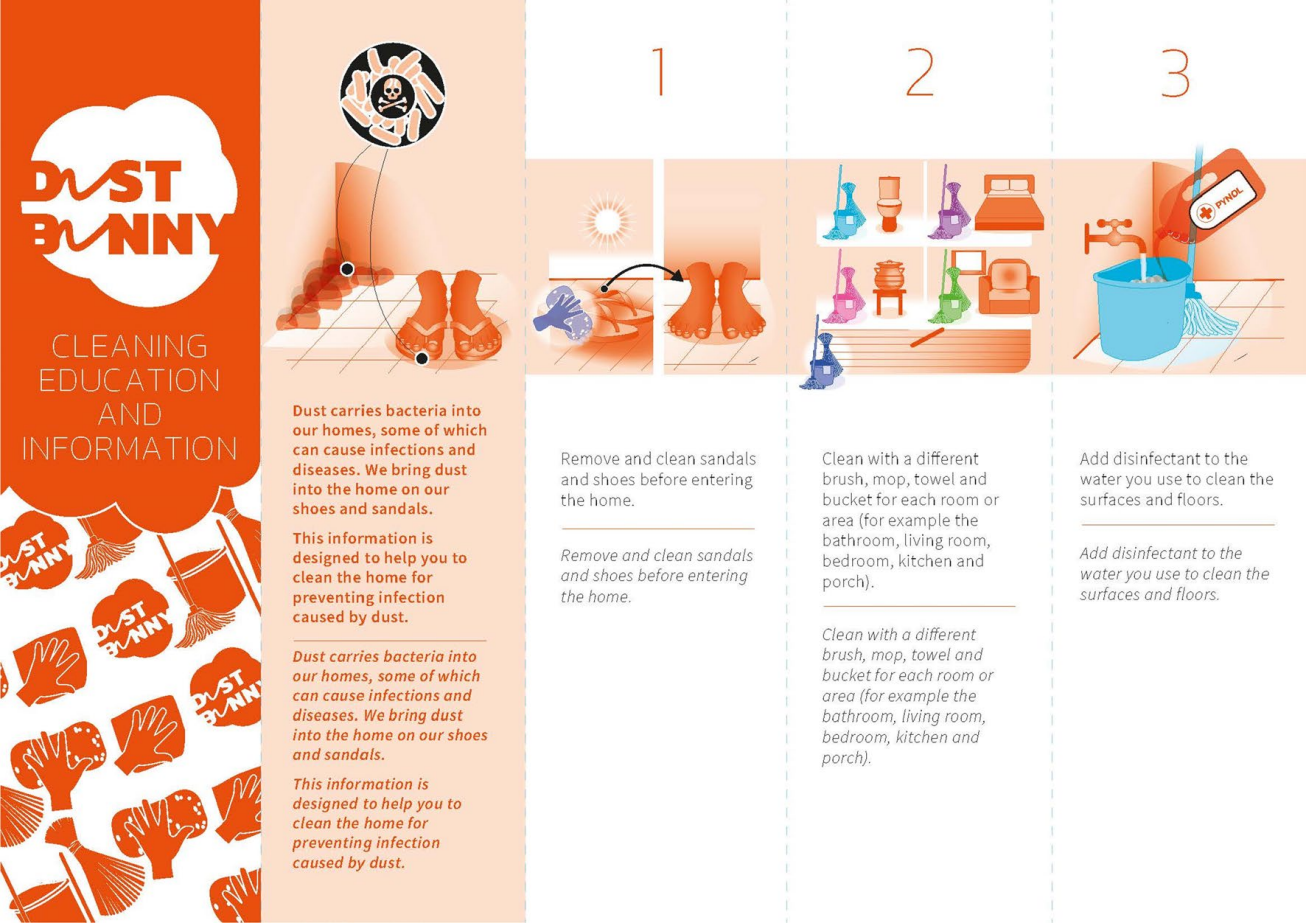
Cleaning education and information material developed as a result of the project.

Doing this, further enhanced the trust between the research team and research participants, as they appreciated that this was a two-way interaction and exchange of knowledge. They could see in-person the benefits of participating in the study and take these lessons learnt back to their household and community, which as we found in the post-intervention interviews, increased acceptance of the intervention and the cleaning education and information material developed as an outcome of the project (see Fig. 6).

The move beyond knowledge sharing in activating knowledge through the user engagement in the research, especially in the co-design workshop was the most important benefit of this approach. It significantly helped research participants develop an understanding of the need and health impact of cleaning at the household level, by discovering and making a link between the Why, the What and the How of home cleaning.

Using co-design for engaging communities for AMR has been used beyond the household environment. For instance, individuals in a community can acquire knowledge about AMR while creating a film on the subject, which they can subsequently utilize in an advocacy initiative aimed at bringing about change^56^. Similarly, in agriculture, comics^57^ can serve as a means of educating about AMR.

Translating and contextualising data visually, especially the microbiology results, made an impact. Being able to visually represent their presence the opportunist pathogens in simulated rooms within our workshop helped significantly towards this. For example, the little boxes shared at the workshop, presenting information on bacterial strains; the risks involved with infection, control methods and their effectiveness, were particularly useful as the participants used these in the various exercises and in developing their cleaning methods/practices. The thought of 18 different bacteria species identified in the study and the risk each of these poses to health elicited some fear in participants. The realization that most bacteria species were susceptible to either bleach or antiseptic treatment or a combination of both, informed some of the cleaning practices developed by the participants, making the invisible visible, highlighting potential routes for contamination.

Firstly, bringing the concept of the unseen microbial world to those unfamiliar with it (making the invisible visible) by being able to see in practice (through the simulated carton boxes and small box bacteria tools) where bacteria were found at participants’ home, enabled them to discover new knowledge and then through the cleaning agreements signed to act upon these. We found that this positively influence new cleaning behaviours and new cleaning practice adoption, which was retained several months after the interventions. People who had participated in the workshop and cleaning interventions become champions for household cleaning in their own communities, as some of the quotes in the result sections reveal. This is very important as evidence suggests that hygiene promotion would contribute to preventing the transmission of resistant bacteria from the household level and everyday life settings, into health care settings, and back into the community^58^.

An unexpected and unintended benefit was that the co-design workshop helped reveal cleaning practice nuances that were not captured by the other research methods previously.

The nuanced information gathered during the workshop revealed new aspects of local cleaning practices and norms, which were then further explored during the post-intervention interviews.

### Limitations

We note several study limitations:

This was a proof-of-concept study. The microbiology sample size was limited to a small number. This was dictated in part by the financial scale of the funder’s call not allowing for more sampling to take place. The dust sampling only took place inside the household. Financial costs permitting we would have liked to take a dust sample from the outside of each household and to account for different seasons (wet and dry). Microbiological analysis of dust samples collected post intervention was not done due to the time and financial limitations of the project. The pre and post intervention dust samples are being sequenced and those results will be presented separately. The study (survey, design ethnography and microbiology analysis) was limited to the greater Accra region. It would have been interesting to explore this in a rural context too.

## Conclusions

The high prevalence of multidrug resistance observed in this study indicate the need for antibiotics surveillance program, not only in hospital settings but also in the household environment. In 2018, Ghana launched the Antimicrobial Use and Resistance Policy and the accompanying comprehensive National Action Plan on Antimicrobial Resistance. Which deals with high level policy and targeted at hospitals and prescribing. There is also an urgent need for targeting of interventions at the household level through education, communication, and surveillance since studies on AMR have mostly focussed on the hospital setting. This study showed that household dust is just one of many human exposure points for bacteria carrying single or multiple resistances and contributes to the household microbiome. In this study only opportunist pathogens were found to carry one or more resistances but still they contribute a threat to human health. The study revealed different nuances in the cleaning patterns and tools across the four socio-economic groups. Social norms and the social judgement of others was found to be a strong motivator affecting local cleaning practices Co-design and community engagement, and enlightenment about this invisible world will drive better cleaning strategies and hopefully reduce the health burden and AMR. It also demonstrated the need and value of providing insights into the behavioural challenges, promoting best practices for public health implementation and improved targeting of interventions at the household level.

## Data Availability

All data (qualitative and quantitative) generated and analysed during this study are included in this paper. Raw qualitative data are not available and will not be shared, as this would compromise the protection of participants’ identity; however, some data could be made available upon a reasonable request from the NMIMR IRB, nirb@noguchi.ug.edu.gh.

## Abbreviations

AMR: Antimicrobial resistance
HIG: High income group
LIG: Lower income group
LMIG: Low-middle income group
LU: Lancaster University
MAR: Multiple antibiotic resistance
MIG: Middle income group
NMIR: Noguchi Memorial Medical Research Institute
